# Supplementary material for: The Spectrum of Mechanism-Oriented Models and Methods for Explanations of Biological Phenomena
Source: Processes (Basel). Author manuscript; Available in PMC 2021 Jul 13. (PMC8277120; doi:10.3390/pr6050056)
Supplement: CPMS rules [file NIHMS1714522-supplement-CPMS_rules.pdf]

Supplementary Materials: The Spectrum of Mechanism-Oriented Models and Methods for Explanations of Biological Phenomena

C. Anthony Hunt, Ahmet Erdemir, William W. Lytton, Feilim Mac Gabhann, Edward A. Sander, Mark K. Transtrum, and Lealem Mulugeta

Role of the Committee on Credible Practice of Modeling and Simulation in Healthcare

Work on the ideas presented began as a project within Committee on Credible Practice of Modeling and Simulation in Healthcare (CPMS) [1].

The importance of semantic and methodology clarity is recognized and further emphasized by a founding cornerstone of the CPMS, which took the charge to adopt consistent terminology in biological modeling and simulation to facilitate clear communication across the multidisciplinary stakeholders within and outside the field. The Committee was formed under the Interagency Modeling and Analysis Group (IMAG; organized by the National Institute of Health) and the Multiscale Modeling (MSM) Consortium [2] to establish credible practice guidelines and identify new areas of research for reliable development and implementation of simulation for healthcare research and application. CPMS recognizes that methods, workflows, software constructs, and simulation experiments described in reports of recent simulation research are becoming increasingly complicated and opaque (e.g., see [3,4]), thus increasing the importance of clarity. Inaccessibility and lack of methodological clarity can act as a repellent to individuals initially interested in a published report for its scientific focus. Inaccessibility can impact different groups of individuals, e.g., 1) researchers outside of computational biology simulation domains, including healthcare professionals, for whom reports of simulation research are intended to be relevant; and 2) students who are initially attracted to the ideas and visions associated with simulation research.

References

1. Credible Practice of Modeling & Simulation in Healthcare. Available online: https://simtk.org/projects/cpms (accessed on 16 March 2018)
2. Interagency Modeling and Analysis Group. Available online: https://www.imagwiki.nibib.nih.gov/ (accessed on 16 March 2018)
3. Meier-Schellersheim, M.; Fraser, I.D.; Klauschen, F. Multiscale modeling for biologists. *WIREs: Syst. Biol. Med.* **2009**, *1*, 4–14, doi:10.1002/wsbm.33
4. Yankeelov, T.E.; An, G.; Saut, O.; Luebeck, E.G.; Popel, A.S.; Ribba, B.; Vicini, P.; Zhou, X.; Weis, J.A.; Ye, K.; Genin, G.M. Multi-scale Modeling in Clinical Oncology: Opportunities and Barriers to Success. *Ann. Biomed. Eng.* **2016**, *44*, 2626–2641, doi:10.1007/s10439-016-1691-6.
